# Reward Learning, Neurocognition, Social Cognition, and Symptomatology in Psychosis

**DOI:** 10.3389/fpsyt.2016.00100

**Published:** 2016-06-14

**Authors:** Kathryn E. Lewandowski, Alexis E. Whitton, Diego A. Pizzagalli, Lesley A. Norris, Dost Ongur, Mei-Hua Hall

**Affiliations:** ^1^Schizophrenia and Bipolar Disorder Program, McLean Hospital, Belmont, MA, USA; ^2^Department of Psychiatry, Harvard Medical School, Boston, MA, USA; ^3^Center for Depression, Anxiety, and Stress Research, McLean Hospital, Belmont, MA, USA

**Keywords:** reward, response bias, cognition, social cognition, psychosis, schizophrenia, bipolar disorder

## Abstract

**Background:**

Patients with psychosis spectrum disorders exhibit deficits in social and neurocognition, as well as hallmark abnormalities in motivation and reward processing. Aspects of reward processing may overlap behaviorally and neurobiologically with some elements of cognitive functioning, and abnormalities in these processes may share partially overlapping etiologies in patients. However, whether reward processing and cognition are associated across the psychoses and linked to state and trait clinical symptomatology is unclear.

**Method:**

The present study examined associations between cognitive functioning, reward learning, and clinical symptomatology in a cross-diagnostic sample. Patients with schizophrenia (SZ; *n* = 37), bipolar I disorder with psychosis (BD; *n* = 42), and healthy controls (*n* = 29) were assessed for clinical symptoms (patients only), neurocognitive functioning using the MATRICS Battery (MCCB) and reward learning using the probabilistic reward task (PRT). Groups were compared on neurocognition and PRT response bias, and associations between PRT response bias and neurocognition or clinical symptoms were examined controlling for demographic variables and PRT task difficulty (discriminability).

**Results:**

Patients with SZ performed worse than controls on most measures of neurocognition; patients with BD exhibited deficits in some domains between the level of patients with SZ and controls. The SZ – but not BD – group exhibited deficits in social cognition compared to controls. Patients and controls did not differ on PRT response bias, but did differ on PRT discriminability. Better response bias across the sample was associated with poorer social cognition, but not neurocognition; conversely, discriminability was associated with neurocognition but not social cognition. Symptoms of psychosis, particularly negative symptoms, were associated with poorer response bias across patient groups.

**Discussion:**

Reward learning was associated with symptoms of psychosis – in particular negative symptoms – across diagnoses, and was predictive of worse social cognition. Reward learning was not associated with neurocognitive performance, suggesting that, across patient groups, social cognition but not neurocognition may share common pathways with this aspect of reinforcement learning. Better understanding of how cognitive dysfunction and reward processing deficits relate to one another, to other key symptom dimensions (e.g., psychosis), and to diagnostic categories, may help clarify shared etiological pathways and guide efforts toward targeted treatment approaches.

## Introduction

Across diagnostic boundaries, patients with psychosis spectrum disorders exhibit substantial deficits in social and neurocognition, as well as hallmark abnormalities in motivation and reward processing. These cognitive and reward processing abnormalities are associated with poor community outcomes, disability, and reduced subjective quality of life (1–3).

Patients with schizophrenia (SZ) exhibit substantial deficits in multiple cognitive processes, including attention, memory, and executive functioning (4). Although cognitive deficits in patients with bipolar disorder (BD) have been found to fall between the level of healthy controls and patients with SZ (5), some studies have shown that BD patients with a history of psychosis exhibit neurocognitive performance similar to patients on the SZ spectrum (6, 7). The study of social cognitive dysfunction across the psychoses has yielded mixed findings: whereas patients with SZ exhibit pronounced deficits in many aspects of social cognition, patients with BD are characterized by more selective deficits (8–10). For instance, patients with BD show deficits in emotion processing and theory of mind (ToM), but not on tasks that assess the ability to anticipate social outcomes or emotional responsivity to social situations (8, 10). Thus, whereas deficits in the so-called “cold” cognition may not differ substantively across psychosis spectrum disorders, deficits in social cognition may differ qualitatively by diagnosis.

There is evidence that many, but not all, aspects of reward processing are abnormal in patients with psychosis. In particular, patients with SZ appear to have deficits in anticipatory but not consummatory pleasure (11, 12); difficulty with value representation but not reward learning *per se* (13); and difficulty with rapid reward-based learning but not gradual learning (3). Patients with BD also exhibit abnormalities in reward processing across various phases of illness (14, 15); specifically, patients in both manic and euthymic states exhibit abnormalities in the modulation of behavior in response to variable reward, impulsivity-related abnormalities in reward processing, and poor integration of reinforcements over time (14, 16).

Whether deficits in reward processing differ categorically by diagnosis or are more closely linked to state and trait clinical symptomatology is unclear. A recent study examining reward learning using a probabilistic reward learning paradigm in a cross-diagnostic sample of patients with SZ or BD (with or without psychosis) found that patients with SZ were characterized by the greatest abnormalities; however, across patient groups, deficits in reinforcement learning were associated with state severity of psychotic symptoms (17). Similarly, abnormalities in anticipatory pleasure were associated with anhedonia (AD) in patients with SZ or major mood disorders (11, 16, 18). However, a recent study found that patients with SZ exhibited lower hedonic experience and behavioral activation than patients with BD despite similar levels of negative affect and trait AD (19). Together, these mixed findings indicate that additional research is needed to clarify the relationships among aspects of reward processing, state clinical symptoms, and diagnostic categories.

Recently, examinations of the intersection of reward processing and cognition have highlighted possible interactions between aspects of reward processing (e.g., reward anticipation and reward-based decision-making) and cognitive performance (e.g., attention, concentration, working memory, and cognitive control) (20–25). In one of the few studies to directly examine associations between cognition and reward processing in patients with SZ or BD, steeper discounting of delayed rewards was associated with poorer intelligence and working memory across patient groups (26). This intersection is further highlighted by research showing that cognitive and reward processing systems share overlapping neurobiological correlates. For example, dopamine is associated both with cognitive processes, such as working memory and cognitive flexibility (27, 28), as well as reward processes, including incentive motivation and reinforcement learning (16, 29). Furthermore, regions such as the anterior cingulate cortex (ACC) have been linked to both reward and cognition (30–33).

Collectively, these data suggest that aspects of reward processing may overlap both behaviorally and neurobiologically with at least some elements of cognitive functioning in healthy adults, and that abnormalities in these processes may share at least partially overlapping etiologies in patients. Given that cognitive dysfunction and reward processing deficits are hallmark symptom dimensions across the psychosis spectrum, gaining a better understanding of how they relate to one another, to other key symptom dimensions (e.g., psychosis), and to diagnostic categories may help clarify shared etiological pathways and guide efforts toward targeted treatment approaches.

To fill gaps in the literature, the aim of this study was to examine associations between neurocognitive functioning, reward learning, and clinical symptomatology in a cross-diagnostic sample of patients with psychosis spectrum disorders and healthy controls. We hypothesized that (1) both reward learning and neurocognitive functioning would be impaired in patients with SZ or BD compared to controls; (2) impairments in reward learning would be associated with poorer neurocognitive and social cognitive functioning across patients with SZ and BD; and (3) impairments in reward learning would be associated with greater psychotic symptom severity.

## Materials and Methods

### Participants

Patients with SZ or schizoaffective disorder (*n* = 37), bipolar I disorder with psychosis (*n* = 42), and healthy controls (*n* = 29) were recruited through the Schizophrenia and Bipolar Disorder Program at McLean Hospital and fliers posted at the hospital. Diagnosis was determined by trained clinicians using the SCID-IV diagnostic interview in conjunction with all available ancillary information from medical records, treatment providers, and family members. Participants were recruited into one of several separate but related studies of cognitive remediation in patients with SZ or BD with psychosis, or a study of clinical and cognitive characterization of patients with psychotic illness. All procedures were approved by the McLean Hospital Institutional Review Board. All subjects gave written informed consent; all procedures were conducted in accordance with the Declaration of Helsinki. Exclusion criteria included history of head trauma with loss of consciousness, history of seizure, alcohol or substance abuse within the last 3 months, and alcohol or substance dependence within the last year. Control participants had no history of a psychiatric diagnosis, no first-degree relatives diagnosed with a psychiatric illness, and no history of alcohol or substance abuse or dependence.

### Materials

Neurocognitive functioning was assessed using the MATRICS Consensus Cognitive Battery [MCCB; (34)]. The MCCB includes 10 tasks across 7 domains, including Processing Speed (Brief Assessment of Cognition in Schizophrenia Symbol Coding, Animal Fluency, Trails A), Attention (Continuous Performance Test), Working Memory (WMS-III Spatial Span, Letter-Number Span), Verbal Learning (Hopkins Verbal Learning Test – Revised), Visual Learning (Brief Visuospatial Memory Test – Revised), Problem Solving (Neuropsychological Assessment Battery), and Social Cognition (Mayer–Salovey–Caruso Emotional Intelligence Test). Total administration time is 60–90 min. The MCCB yields 10 subscale scores, 7 domain scores, and a composite score.

Reward learning was probed using the probabilistic reward task [PRT; (35); modified after Tripp and Alsop (36)]. Grounded within signal-detection theory, the PRT provides an index of a person’s ability to modulate behavior based on reinforcement. In this computer-administered task, participants are presented with several trials in which schematic cartoon faces with two eyes and a nose are presented on the monitor. Next, a horizontal straight line (the mouth) is presented quickly (100 ms) and participants are required to indicate via key press whether the mouth was long (11.5 mm) or short (13 mm). The task consists of three blocks of 100 trials, and on 40% of correct trials participants receive a monetary reward of 20 cents (“Correct! You won 20 cents”). Long and short mouths were presented at equal frequency, however, unbeknownst to participants, correct identification of one stimulus (“rich stimulus”) was rewarded three times more frequently than the other stimulus (“lean stimulus”). Among healthy controls, this asymmetrical reinforcement ratio results in a behavioral response bias toward the rich stimulus (35), and the strength of this bias is reflective of an individual’s sensitivity to reward. As data were compiled from separate but related studies, two different versions of the PRT were used, one in which long and short mouths were 1.5 mm different in length (11.5 versus 13 mm) and one in which the mouths were 1 mm different in length (9 and 10 mm). Groups differed significantly by task version [χ^2^_(2)_ = 31.08; *p* < 0.001], as all controls received the same version of the task. Patient groups did not differ in terms of distribution of task version [χ^2^_(2)_ = 2.22; *p* = 0.14].

The Positive and Negative Syndrome Scale [PANSS; (37)], the Young Mania Rating Scale [YMRS; (38)], and the Montgomery-Asberg Depression Rating Scale [MADRS; (39)] were used to evaluate current psychotic and mood symptoms; the Mood and Anxiety Symptom Questionnaire [MASQ; (40)] was used to evaluate AD, anxious arousal (AA), general distress related to anxiety (GDA), and general distress related to depression (GDD); hedonic tone was measured using the Snaith–Hamilton Pleasure Scale [SHPS; (41)]. Community functioning was evaluated using an abbreviated version of the Multnomah Community Ability Scale [MCAS; (42)]. We administered a modified version, focusing on items that directly assessed domains of community functioning, including independence in daily living, social involvement and interest, and instrumental role functioning. This version included 11 items scored 1–5 (higher scores indicate better functioning) for a total of 55 points. The Hollingshead Four Factor Index of Social Status^1^ was used to calculate parental socioeconomic status (SES). Information about medication at the time of assessment was obtained from participants and chlorpromazine (CPZ) equivalents were calculated based on the recommendations of Baldessarini (43).

### Procedures

Cognitive and clinical assessments were typically conducted in single sessions lasting ~2 h. PRT administration lasted approximately 20–30 min, and was either performed at the end of the cognitive and clinical assessments or in a separate session. Procedures across all studies were standardized and the same study staff completed these assessments.

Scores from the MCCB were age normed using the provided scoring software. All subscale, domain, and composite scores are presented as normed T scores with a mean of 50 and SD of 10.

The PRT data were subject to a quality control check where trials with reaction time <150 ms or >2500 ms were excluded, and participants with <55% accuracy, more than 10% outlier trials, or a rich:lean reward ratio lower than 2.5:1 (as a result of poor accuracy/slow reaction time) were excluded from analysis. Signal-detection analysis was used to compute response bias (the tendency to bias responding to the rich stimulus) as well as discriminability (the ability to distinguish between the two mouth sizes), for each block of the PRT using the following formulae:

$$\text{Response bias: log }b=\frac{1}{2}\log\left(\frac{{\text{Rich}}_{\text{correct}}⋆{\text{Lean}}_{\text{incorrect}}}{{\text{Rich}}_{\text{incorrect}}⋆{\text{Lean}}_{\text{correct}}}\right)$$

$$\text{Discriminability: log }d=\frac{1}{2}\log\left(\frac{{\text{Rich}}_{\text{correct}}⋆{\text{Lean}}_{\text{correct}}}{{\text{Rich}}_{\text{incorrect}}⋆{\text{Lean}}_{\text{incorrect}}}\right)$$

To compute response bias and discriminability for cases that had a 0 in the formula, 0.5 was added to each cell of the matrix (44).

### Statistical Approach

Groups were compared on neurocognitive and PRT performance using ANOVA and pairwise *t*-tests with Bonferroni correction. Partial correlations examining the association of neurocognitive, social cognitive, and reward learning outcomes with clinical measures controlling for age and gender were conducted across the patient groups and by diagnosis. Associations between PRT and MCCB were examined using correlation, and then linear regression controlling for demographic variables and PRT task difficulty. The association of reward learning and symptom severity was examined in regression models using PRT as a predictor of mania, depression, positive and negative symptoms, and anxiety after accounting for the effects of demographic confounders and PRT discriminability.

## Results

Groups differed on several demographic variables, including education (SZ < BD, HC) and gender; groups did not differ on level of parental SES (see Table 1). Patient groups differed on several measures of state clinical severity, including PANSS Positive, Negative, and Total scores, with patients with SZ exhibiting higher PANSS scores than BD patients; patients did not differ in terms of state mania or depression (see Table 1). Note that overall symptom severity in both groups was relatively low. Patients differed from controls on the SHPS as well as MASQ AA, AD, GDA, and GDD scores, but did not differ from each other. Patients did not differ from each other on the MCAS community functioning measure.

**Table 1:** **Demographic and clinical variables by diagnosis**.

	SZ (*n* = 37)	BD (*n* = 42)	HC (*n* = 29)	Test statistic
**Demographic**				
Age	35.0 (11.9)	29.6 (8.4)	31.0 (10.0)	*F*_(2, 105)_ = 2.88
Education	14.1 (2.3)	15.6 (1.7)	16.1 (2.1)	F_(2, 104)_ = 8.85***
Gender (% female)	30%	55%	59%	Chi2 = 7.01*
Ethnicity (% Caucasian)	89%	98%	100%	Chi2 = 5.09
Parental SES^a^	55.4 (11.9)	52.0 (11.5)	48.6 (10.3)	*F*_(2, 47)_ = 0.94
**Clinical**				
CPZ	370.5 (333.0)	173.0 (213.6)	N/A	*t*_(55)_ = 2.73**
# hosp.	8.3 (6.4)	5.3 (8.1)	N/A	*t*_(27)_ = 1.04
YMRS	7.0 (6.4)	5.6 (4.9)	N/A	*t*_(57)_ = 0.95
MADRS	8.9 (8.2)	10.3 (8.3)	N/A	*t*_(59)_ = -0.66
PANSS P	13.9 (5.8)	10.5 (4.0)	N/A	*t*_(57)_ = 2.68**
PANSS N	12.5 (4.9)	10.3 (3.0)	N/A	*t*_(57)_ = 2.14*
PANSS G	27.2 (7.4)	24.2 (6.5)	N/A	*t*_(57)_ = 1.67
PANSS Total	54.2 (14.9)	44.7 (10.7)	N/A	*t*_(57)_ = 2.88**
MCAS	47.0 (4.5)	49.0 (3.8)	N/A	*t*_(27)_ = -1.82
SHPS	1.76 (2.56)	1.87 (2.62)	0.14 (0.44)	*F*_(2, 73)_ = 6.06**
MASQ AA	24.6 (8.3)	23.5 (7.6)	19.6 (5.3)	*F*_(2, 72)_ = 3.56*
MASQ AD	59.5 (21.2)	57.7 (18.6)	44.4 (9.2)	*F*_(2, 72)_ = 6.52**
MASQ GDA	18.8 (8.4)	17.6 (5.5)	14.1 (4.0)	*F*_(2, 72)_ = 4.30*
MASQ GDD	22.0 (11.0)	23.6 (8.4)	15.0 (4.5)	*F*_(2, 72)_ = 9.18***

*^a^Parental SES was available for a subset of participants (SZ: *n* = 16; BD: *n* = 7; HC: *n* = 27). When patient groups were combined (*n* = 23) and compared to controls (*n* = 27) the results remained unchanged (*t* = −0.41, *p* > 0.05)*.

*p < 0.05.

**p < 0.01.

***p < 0.001.

### Cognitive Functioning

**Figure 1:**
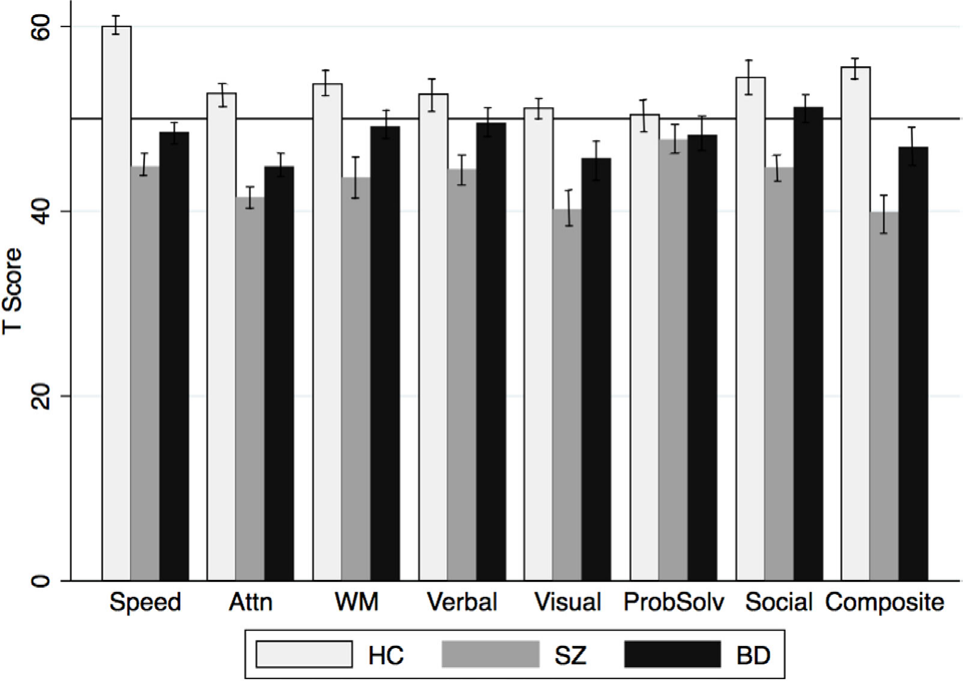
**MCCB domain scores by group**. MCCB domain (T scores) by diagnosis. Speed, Processing Speed; Attn, Attention; WM, Working Memory; Verbal, Verbal Learning and Memory; Visual, Visual Learning and Memory; ProbSolv, Problem Solving; Social, Social Cognition; Composite, Cognitive Composite.

ANOVA results showed that groups differed significantly on the MCCB Social Cognition domain and on all neurocognitive domains except Problem Solving (see Table 2; Figure 1). Pairwise comparisons of the significant findings (i.e., all domains except Problem Solving) with Bonferroni correction revealed that patients with SZ performed worse than HC on all neurocognitive and social cognitive domains, and the Cognitive Composite (*p* < 0.05 to *p* < 0.001; Cohen’s *d* = 0.74–1.61). Patients with BD performed worse than HC on Processing Speed [*t*_(69)_ = 4.19; *p* < 0.001; Cohen’s *d* = 1.04], Attention [*t*_(69)_ = 3.32; *p* < 0.01; Cohen’s *d* = 0.81], and the Composite [*t*_(69)_ = 3.75; *p* < 0.01; Cohen’s *d* = 0.93]. Patients with BD and SZ differed on Working Memory [SZ < BD; *t*_(76)_ = −2.31 *p* < 0.05; Cohen’s *d* = 0.51], Social Cognition [SZ < BD: *t*_(76)_ = −3.13, *p* < 0.05; Cohen’s *d* = 0.70], and the overall neurocognitive Composite [SZ < BD: *t*_(76)_ = −2.81, *p* < 0.01; Cohen’s *d* = 0.64]. A series of partial correlations examining associations between clinical variables (YMRS, MADRS, PANSS Positive, PANSS Negative, PANSS General, and PANSS Total) and cognitive outcomes correcting for age and gender in the total patient sample and separately by diagnosis showed that clinical symptoms were not significantly associated with any neurocognitive domain, social cognition, or the composite score.

**Table 2:** **Cognitive and reward processing scores by diagnosis**.

	SZ (*n* = 37)	BD (*n* = 42)	HC (*n* = 29)	Test statistic
**Cognitive**				
Processing	44.8 (10.1)	48.5 (12.7)	60.0 (9.1)	*F*_(2, 104)_ = 15.71**
Attention	41.4 (11.8)	44.8 (10.5)	52.7 (8.9)	*F*_(2, 104)_ = 9.51**
WM	43.6 (11.6)	49.1 (9.7)	53.8 (8.3)	*F*_(2, 104)_ = 8.44**
Verbal	44.4 (11.3)	49.5 (9.3)	52.6 (11.0)	*F_(_*_2, 104)_ = 5.14*
Visual	40.1 (10.8)	45.6 (11.0)	51.1 (6.9)	*F*_(2, 104)_ = 9.68**
Prob Solv	47.7 (10.1)	48.2 (9.5)	50.4 (9.6)	*F*_(2, 104)_ = 0.67
Social	44.7 (9.5)	51.2 (9.0)	54.4 (10.7)	*F*_(2, 104)_ = 8.83**
Composite	39.8 (11.5)	46.9 (10.7)	55.5 (7.6)	*F*_(2, 104)_ = 18.92**
**PRT**				
RB Block 1	0.11 (0.21)	0.07 (0.17)	0.15 (0.28)	*F*_(2, 96)_ = 1.19
RB Block 2	0.14 (0.23)	0.10 (0.15)	0.16 (0.24)	*F*_(2, 97)_ = 0.92
RB Block 3	0.17 (0.25)	0.14 (0.20)	0.16 (0.30)	*F*_(2, 97)_ = 0.16
RB Main	0.15 (0.20)	0.11 (0.15)	0.16 (0.23)	*F*_(2, 96)_ = 0.63
RB Total	0.13 (0.19)	0.10 (0.13)	0.16 (0.21)	*F*_(2, 96)_ = 0.90
Discrim.	0.67 (0.39)	0.57 (0.25)	0.92 (0.34)	*F*_(2, 96)_ = 9.95**

*p < 0.01.

**p < 0.001.

### Reward Processing

**Figure 2:**
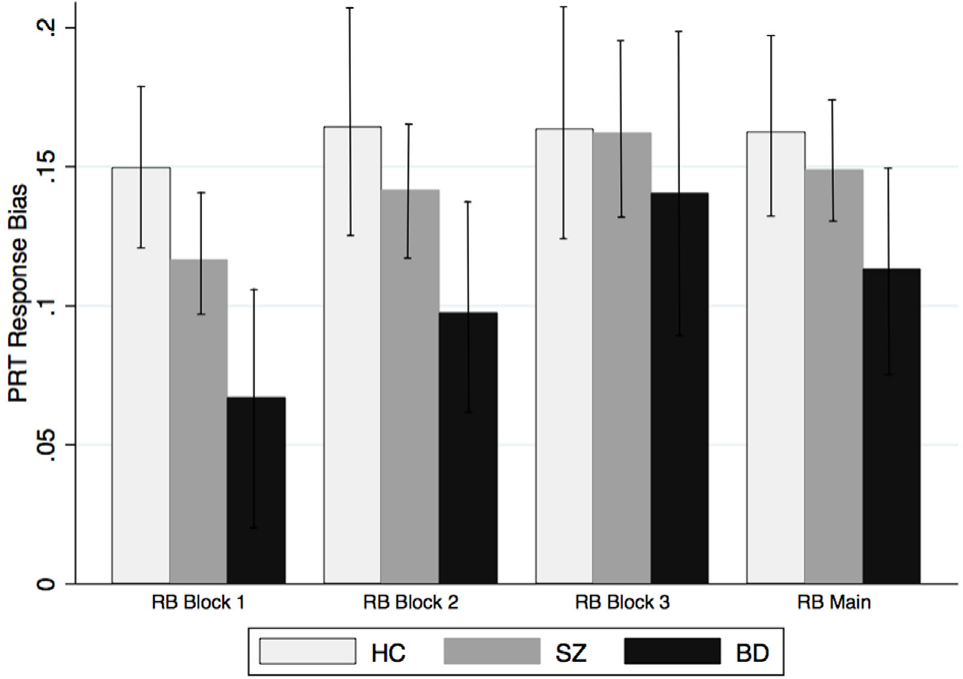
**PRT response bias by group**. PRT response bias scores by diagnosis across Block 1, Block 2, and Block 3, and response bias aggregated across Blocks 2 and 3 (RB Main).

Probabilistic reward task data from several participants were excluded after quality control checks were performed blind to group assignment (below-chance responding, poor rich reward count, and too many outliers; SZ: *n* = 6; BD: *n* = 4; HC: *n* = 0). PRT scores did not differ by test version across the total sample or by diagnosis. Results indicated that when examined separately across the three blocks of the task, in the latter portion of the task only (RB main = mean RB in blocks 2 and 3) or across the entire task (RB total), PRT response bias did not differ significantly between patients and controls, or by diagnosis (all *p*s > 0.05; Table 2). Partial correlations conducted separately and by group accounting for the effects of age and gender showed no effect of these variables on response bias. Although not statistically significant, the BD group appeared to acquire a response bias more slowly than the HC or SZ groups (Figure 2). A main effect of *Group* emerged for PRT discriminability, a measure of overall task difficulty; specifically, patients with SZ and BD exhibited lower discriminability scores than the HC group [*t*_(63)_ = 3.39; *p* < 0.01 and *t*_(69)_ = 4.86; *p* < 0.001, respectively]; patients did not differ from each other [*t*_(76)_ = 0.32, *p* = 0.75]. Results involving response bias did not change after controlling for overall discriminability scores. As noted above, current substance abuse and history of substance dependence within the past year were exclusion criteria; however, data on lifetime history of substance abuse or dependence and lifetime and current tobacco and cannabis use patterns were available in a subset of patients. Thus, in order to examine the potential effects of smoking and other substance use on PRT response, we examined the effects of lifetime history of substance abuse (*n* = 44), lifetime history of tobacco smoking (*n* = 59) and current smoking status (*n* = 48), and lifetime and current cannabis use (*n* = 39) using partial correlation. After controlling for age and sex, none of our measures of smoking or other substance use was correlated with PRT response bias.

### Relationship between Reward Processing and Cognition

Correlations among PRT and MCCB variables showed that neither Social Cognition nor any of the neurocognitive domains was correlated with PRT response bias. Across groups, PRT discriminability was significantly correlated with most neurocognitive domains, including Processing Speed (*r* = 0.34, *p* < 0.001), Attention (*r* = 0.44, *p* < 0.001), Working Memory (*r* = 0.20, *p* < 0.05), Verbal (*r* = 0.40, *p* < 0.001), Visual (*r* = 0.35, *p* < 0.001), and the Cognitive Composite (*r* = 0.43, *p* < 0.001). In order to correct for multiple correlational analyses, we utilized a procedure adjusting for multiple correlations following the recommendations in Sankoh et al. (45). In this method, within groupings of correlational analyses *p*-values are adjusted by accounting for the number of correlations and the mean correlation among all variables excluding the kth correlation. All significant correlations survived adjustment except Working Memory. Linear regression predicting MCCB measures by PRT response bias after accounting for the effects of sex, education, PRT discriminability, and diagnosis revealed that response bias was a significant predictor of Social Cognition (β = −10.78; *t* = −2.13, *p* < 0.05), such that lower response bias was associated with higher Social Cognition scores. The model did not predict any MCCB neurocognitive domain scores.

### Relationship between Reward Processing and Clinical Symptom Severity

PRT response bias was significantly correlated with several clinical scales, including PANSS Positive (*r* = −0.28, *p* < 0.05), PANSS Negative (*r* = −0.30 *p* < 0.05), PANSS General (*r* = −0.36, *p* < 0.01), and PANSS Total (*r* = −0.30, *p* < 0.05), with higher response bias being associated with less severe symptomatology. Partial correlations confirmed that these correlations remained significant when controlling for age and sex (all *p*s < 0.05). Note that using the above multiple comparisons correction only the correlation between PANSS General and PRT response bias remained significant [*p(corr)* < *0.05*]; PANSS Negative and PANSS Total scores showed trend-level significance [*p(corr)* = *0.05*]. PRT response bias was not correlated with CPZ, mania severity, depression severity, state anxiety, or community functioning. Partial correlations run separately by diagnosis showed that in the BD group, higher PRT response bias was associated with lower PANSS General scores after accounting for the effects of age and sex (*r* = −0.36; *p* = 0.05). There were no other significant associations between clinical symptoms and PRT scores, likely due to small sample size; however, the magnitude of the correlations was similar to the total group, and comparable in the SZ and BD groups for most measures (PANSS Total: *r* = −0.29, *r* = −0.28; PANSS Negative: *r* = −0.28; *r* = −0.30; PANSS General: *r* = −0.33, *r* = −0.36, respectively). PANSS Positive and PRT response bias showed different associations by diagnosis (SZ: *r* = −0.33; BD: *r* = −0.09). The range of PANSS scores was comparable by group (SZ: 7–26; BD: 7–20). PRT discriminability was not correlated with any clinical measure or CPZ equivalents. When negative symptoms and positive symptoms were included in a regression model in separate steps, inclusion of positive symptoms after accounting for negative symptoms did not contribute significantly to the prediction of PRT response bias; the reverse was also true.

Two sets of regression models were used to determine whether PRT response bias and cognitive functioning were predictors of positive and negative symptom severity in the patient groups after accounting for age, education, PRT discriminability, and social or neurocognitive score. For the first regression model, age, education, PRT discriminability, PRT response bias, and the Cognitive Composite were entered as predictors; Social Cognition replaced the Composite in the second model. Response bias was a significant predictor of PANSS Positive (β = −9.96, *p* < 0.05), PANSS Negative (β = −6.86, *p* < 0.05), PANSS General (β = −16.55, *p* < 0.01), and PANSS Total (β = −27.63, *p* < 0.05); neither the Cognitive Composite nor Social Cognition were significant predictors in any model.

## Discussion

In the present study, we examined neurocognition, social cognition, and reward learning in patients with SZ, BD-I with psychosis, and HC, and the association between cognitive functioning, reward learning, and clinical symptoms. As we hypothesized and consistent with previous findings, patients with BD and SZ exhibited significant deficits in multiple domains of neurocognition compared to HC, with patients with BD performing between the level of HC and patients with SZ on most measures. Conversely, patients with BD did not differ from HC on the MSCEIT Social Cognition measure, and both patients with BD and HC performed significantly better than patients with SZ. This suggests that, although neurocognitive functioning is impaired across the psychosis spectrum, some aspects of social cognition may be intact in patients with BD and may differentiate patients with BD from patients with SZ spectrum disorders. These findings support a growing literature demonstrating that deficits in the aspects of social cognition measured by the MSCEIT may represent a unique phenotype of SZ but not psychotic mood disorders (10, 46). While many major symptom domains, such as neurocognition are now well known to cut across the “Kraepelinian divide,” some details of these symptoms and their expression may differ by diagnosis or illness dimension in meaningful ways. For instance, even within the domain of neurocognition in psychosis, evidence of distinct premorbid and prodromal trajectories by diagnosis (7) may reflect subtle but meaningful variation in etiological pathways, course mediators, or treatment indications.

Contrary to our hypothesis, neither patient group differed from HC on response bias in the PRT reward learning paradigm. Although some aspects of reward learning appear to be impaired in patients with psychosis, in our sample patients did not differ from healthy controls, suggesting that patients were able to successfully modify behavior as a function of reinforcement. This is consistent with recent findings from Hager and colleagues (47), who reported no difference between patients with SZ and healthy controls in neural activation in the context of a differentially rewarded n-back task, indicating that patients were able to successfully use reward processing to maximize cognitive performance.

Across groups, PRT response bias was not significantly correlated with any neurocognitive domain, but was associated with Social Cognition. This finding suggests that reward learning may be more closely tied with “hot” social cognitive processes as opposed to “cold” cognition. Interestingly, better reward learning on the PRT was actually associated with poorer social cognitive processing. One possible explanation for this unexpected finding is that the response choices in the MSCEIT task often include seemingly socially desirable but less nuanced selections. Reponses are given on a Likert scale, such that repeated selection of socially desirable choices may lead to an overall lower score, confounding the reinforcing effects of socially desirable decision-making with social cognitive processing and perspective taking. However, this finding needs further exploration. A *post hoc* correlational analysis revealed that Social Cognition was not correlated with any neurocognitive domain except Problem Solving (*r* = 0.20, *p* < 0.05) and that neurocognitive domains showed a strong pattern of inter-correlation, with most domains modestly to moderately correlated with most or all other domains. This finding suggests that social cognition as measured by the MSCEIT taps a unique domain of brain function, which does not overlap significantly with most other aspects of neurocognition but may share some common pathways with reward learning.

The present findings linking reward processing and social cognition but not neurocognition suggest dissociable mechanisms of effect for these hallmark symptom dimensions. While social cognition is often considered a subdomain of neurocognition, it may rely on at least partially separable pathways that are differentially associated with aspects of reward processing. Recent reports suggest potential common pathways between reward processing and social cognition both regionally and at the circuit level. Aspects of both social cognition (e.g., ToM) and reward and motivation are associated with brain regions, including anterior cingulate, amygdala, and areas of the prefrontal cortex. Also, connections between regions of the dorsolateral and ventral medial prefrontal cortex and anterior cingulate are implicated in both reward-based learning and aspects of social cognition (48). Patients with SZ show decreased BOLD activation and reduced gray matter volume in medial frontal and cingulate regions during both reward learning (49) and social reasoning and ToM tasks (50). A recent study found that medial PFC activation during a ToM task mediated the relationship between social AD and social functioning in patients with SZ (51), suggesting a possible shared neural pathway between ToM, AD, and social functional outcomes in SZ. Collectively, these findings highlight associations between medial PFC and ACC activation and the relationship of these connections to reward learning, motivation, and AD, suggesting a neurobiological pathway linking these constructs, which may be abnormal in patients with SZ and related disorders.

In terms of clinical symptoms, response bias was associated with psychotic symptom severity across patient groups, including positive symptoms, negative symptoms, and general symptoms, although after multiple comparisons correction only the association with general symptoms remained significant, with negative and total scores at the trend level. State mood and anxiety symptoms were not associated with reward learning measures. Substantial literature supports the association between negative symptoms and aspects of reward and motivation; our findings indicate that reward learning was associated negative symptoms and general symptoms of psychosis across diagnoses, suggesting that these associations reflect dimensions that cut across diagnostic boundaries. However, response bias was only associated with positive symptoms in patients with SZ; this differential pattern of associations suggests that the relationship of response bias with positive symptoms of psychosis may differ by diagnosis, perhaps based on differences in the nature of positive symptoms in these patient groups. While outside the scope of this work, careful evaluation of positive symptom items or factors that predict response bias would help clarify these relationships.

The present study has several limitations; most notably, only one measure of reward processing (a behavioral measure of reward learning) was included, limiting our ability to examine multiple aspects of reward processing and their differential associations with neuro- and social cognitive performance and clinical symptoms. Studies using multiple measures of reward processing tapping putatively different domains will help clarify the relationships among specific aspects of these key symptom domains. Additionally, two slightly different PRT versions were used in this study. However, PRT version was not associated with response bias in our sample. Across studies, subjects were excluded if they endorsed current substance abuse or recent dependence; we are, therefore, unable to examine associations between current substance misuse and reward and cognition. However, lifetime history of abuse or dependence was not associated with PRT response bias. In terms of smoking, data regarding history of smoking were available for only a subset of our participants; however, in this subset history of smoking was not associated with response bias. Information on medical comorbidity was not collected. Per our exclusion criteria, patients with histories of head trauma, seizure disorder, or other major neurological/medical illnesses that may suggest a non-idiopathic psychosis were not eligible to participate. Our sample was young, with a mean age of approximately 32 years, suggesting low risk for major medical burden that might significantly impact cognitive status. However, we cannot rule out the possibility that medical conditions affected participants’ cognitive status. Lastly, our participants were largely stable outpatients at the time of testing, limiting our ability to generalize findings to patients with more acute exacerbations, or to stratify our samples based on SZ subtypes or current polarity in our BD sample. The relationships among cognition, symptoms, and reward during stability/euthymia versus exacerbation and the extent to which associations converge or decouple with symptom fluctuation should be a focus of future work.

In sum, reward learning was associated with symptoms of psychosis – in particular negative symptoms – across diagnoses, and was predictive of scores on the MSCEIT Social Cognition task. Reward learning was not associated with neurocognitive performance, however, suggesting that social cognition but not neurocognition may share common pathways with this aspect of reinforcement learning. Better understanding of the associations among reward and cognition will clarify the pathways that are shared and distinct in these key symptom dimensions, and may hasten the development of interventions to target these domains.

## Author Contributions

KL contributed to all aspects of manuscript preparation, study design, data analysis, and interpretation. AW contributed to data analysis, interpretation, and manuscript preparation; DP was involved in interpretation of results and manuscript preparation; LN was involved in data collection and management, analysis, and manuscript preparation; DO was involved in study design and manuscript preparation; MH was involved in design and development of the research project, interpretation of findings, and manuscript preparation.

## Conflict of Interest Statement

Dr. DP has received consulting fees from Otsuka America Pharmaceutical, BlackThorn Therapeutics, and Pfizer, for activities unrelated to the current research. Dr. DO has received research support from Roche Genentech. KL, AW, and LN have no financial or commercial disclosures to report in relation to this work.

## Footnotes

^1^Hollingshead AB. *Four Factor Index of Social Status*. (1975). Unpublished working paper.
